# A multicenter clinical epidemiology of pediatric pneumococcal meningitis in China: results from the Chinese Pediatric Bacterial Meningitis Surveillance (CPBMS) 2019–2020

**DOI:** 10.3389/fcimb.2024.1353433

**Published:** 2024-03-15

**Authors:** Caiyun Wang, Hongmei Xu, Gang Liu, Jing Liu, Hui Yu, Biquan Chen, Guo Zheng, Min Shu, Lijun Du, Zhiwei Xu, Lisu Huang, Haibo Li, Sainan Shu, Yinghu Chen

**Affiliations:** ^1^ Department of Infectious Disease, Children’s Hospital, Zhejiang University School of Medicine, National Clinical Research Center for Child Health, National Children’s Regional Medical Center, Hangzhou, Zhejiang, China; ^2^ Department of Infectious Disease, Children’s Hospital of Chongqing Medical University, Chongqing, China; ^3^ Department of Infectious Diseases, Key Laboratory of Major Diseases in Children, Ministry of Education, Beijing Children’s Hospital, Capital Medical University, National Center for Children’s Health, Beijing, China; ^4^ Research Unit of Critical Infection in Children, Chinese Academy of Medical Sciences, Beijing, China; ^5^ Department of Infectious Disease, Hunan Children’s Hospital, Changsha, Hunan, China; ^6^ Department of Infectious Disease, The Children’s Hospital of Fudan University, Shanghai, China; ^7^ Department of Infection, Anhui Province Children’s Hospital, Hefei, Anhui, China; ^8^ Department of Neurology, Children’s Hospital of Nanjing Medical University, Nanjing, Jiangsu, China; ^9^ Department of Pediatrics, West China Second University Hospital, Sichuan University/West China Women’s and Children’s Hospital, Chengdu, Sichuang, China; ^10^ Department of Neurology, Children’s Hospital of Shanxi, Taiyuan, Shanxi, China; ^11^ Pediatric Inpatient Ward, The 2nd Affiliated Hospital and Yuying Children’s Hospital of Wenzhou Medical University, Wenzhou, Zhejiang, China; ^12^ Department of Infectious Disease, Xinhua Hospital Affiliated to Shanghai Jiao Tong University School of Medicine, Shanghai, China; ^13^ Outpatient Department of Pediatrics, The First Hospital of Jilin University, Changchun, Jilin, China; ^14^ Department of Pediatric Infection and Gastroenterology, Tongji Hospital, Tongji Medical College of Huazhong University of Science and Technology, Wuhan, Hubei, China

**Keywords:** *S. pneumoniae*, meningitis, epidemiology, multicenter study, pediatric

## Abstract

**Objective:**

To analyze the clinical epidemiological characteristics including clinical features, disease prognosis of pneumococcal meningitis (PM), and drug sensitivity of *S. pneumoniae* isolates in Chinese children.

**Methods:**

A retrospective analysis was performed on the clinical, laboratory microbiological data of 160 hospitalized children less than 15 years of age with PM from January 2019 to December 2020 in 33 tertiary hospitals in China.

**Results:**

A total of 160 PM patients were diagnosed, including 103 males and 57 females The onset age was 15 days to 15 years old, and the median age was 1 year and 3 months. There were 137 cases (85.6%) in the 3 months to <5 years age group, especially in the 3 months to <3 years age group (109 cases, 68.2%); *S. pneumoniae* was isolated from cerebrospinal fluid (CSF) culture in 95(35.6%), and 57(35.6%) in blood culture. The positive rates of *S. pneumoniae* detection by CSF metagenomic next-generation sequencing (mNGS)and antigen detection method were 40.2% (35/87) and 26.9% (21/78). Fifty-five cases (34.4%) had one or more predisposing factors of bacterial meningitis; and 113 cases (70.6%) had one or more extracranial infection diseases Fever (147, 91.9%) was the most common clinical symptom, followed by vomiting (61, 38.1%) and altered mental status (47,29.4%). Among 160 children with PM, the main intracranial imaging complications were subdural effusion and (or) empyema in 43 cases (26.9%), hydrocephalus in 24 cases (15.0%), cerebral abscess in 23 cases (14.4%), intracranial hemorrhage in 8 cases (5.0%), and other cerebrovascular diseases in 13 cases (8.1%) including encephalomalacia, cerebral infarction, and encephalatrophy. Subdural effusion and (or) empyema and hydrocephalus mainly occurred in children < 1 years old (90.7% (39/43) and 83.3% (20/24), respectively). 17 cases with PM (39.5%) had more than one intracranial imaging abnormality. *S. pneumoniae* isolates were completely sensitive to vancomycin (100.0%, 75/75), linezolid (100.0%,56/56), ertapenem (6/6); highly sensitive to levofloxacin (81.5%, 22/27), moxifloxacin (14/17), rifampicin (96.2%, 25/26), and chloramphenicol (91.3%, 21/23); moderately sensitive to cefotaxime (56.1%, 23/41), meropenem (51.1%, 23/45) and ceftriaxone (63.5, 33/52); less sensitive to penicillin (19.6%, 27/138) and clindamycin (1/19); completely resistant to erythromycin (100.0%, 31/31). The cure and improvement rate were 22.5% (36/160)and 66.3% (106/160), respectively. 18 cases (11.3%) had an adverse outcome, including 6 cases withdrawing treatment therapy, 5 cases unhealed, 5 cases died, and 2 recurrences. *S. pneumoniae* was completely susceptible to vancomycin (100.0%, 75/75), linezolid (100.0%, 56/56), and ertapenem (6/6); susceptible to cefotaxime, meropenem, and ceftriaxone in the order of 56.1% (23/41), 51.1% (23/45), and 63.5 (33/52); completely resistant to erythromycin (100.0%, 31/31).

**Conclusion:**

Pediatric PM is more common in children aged 3 months to < 3 years old. Intracranial complications mostly occur in children < 1 year of age with fever being the most common clinical manifestations and subdural effusion and (or) empyema and hydrocephalus being the most common complications, respectively. CSF non-culture methods can facilitate improving the detection rate of pathogenic bacteria. More than 10% of PM children had adverse outcomes. *S. pneumoniae* strains are susceptible to vancomycin, linezolid, ertapenem, levofloxacin, moxifloxacin, rifampicin, and chloramphenicol.

## Introduction

*Streptococcus pneumoniae* (*S. pneumoniae*) is the leading causative agent of bacterial meningitis in children more than 3 months of age (Guo et al., 2016; Li et al., 2018), which is an important pathogen of death of children (Organization, 2019). Due to the lack of domestic public awareness of the lack of knowledge related to *S. pneumoniae* infections, low vaccination coverage with geographic variations (Organization WH., 2021), difficulties in rapid and accurate pathogenetic testing, lack of an effective surveillance system, and changing patterns of pathogen resistance, the Global Burden of Disease Study estimated that in 2016, the number of Chinese pneumococcal meningitis (PM) deaths amounted to 606 cases, with a mortality rate of 0.04/100,000 (Collaborators GCoD, 2017). The mortality rate of PM is nearly 20%-30% (Li et al., 2018), and 30%-52% of cases may develop neurologic long-term sequel (Hénaff et al., 2017), with the most severe long-term effects on survivors (GBD 2016 Brain and Other CNS Cancer Collaborators, 2019; GBD 2016 Neurology Collaborators, 2019), seriously threatening the health of children in China. Therefore, a complete understanding of the epidemiological features, clinical characteristics, common complications, and antimicrobial susceptibility of PM in children in China, as well as early and rational treatments are key to improving prognosis. From January 2019 to December 2020, we initiated a nationwide study in 33 tertiary hospitals in Grade A to collect clinical and laboratory data on PM in hospitalized children to learn understand the season of onset, age of onset, clinical features, common intracranial imaging complications, prognosis and recurrence, and antimicrobial susceptibility of PM in Chinese children, to reflect more comprehensively the epidemiological features of PM and the trend of disease change in China’s pediatric PM, and dynamically optimizing the adjustment of prevention and treatment strategies.

## Materials and methods

### Study design and procedures

From 2019 to 2020, we conducted a national multicenter retrospective study of children (<15 years of age) diagnosed with PM across seven geographical divisions of China. A total of 33 tertiary Grade A hospitals in 23 provinces (27 cities) participated in this study. Of the 33 collaborating hospitals, 13 are in East China, 6 in North China, 4 each in Central and Northwest China, 3 in Southwest China, 2 in South China, and 1 in Northeast China.

### Patient inclusion and exclusion criteria

All patients enrolled met the standards of PM diagnosis established by the World Health Organization (WHO) (Organization WH., 2003). The PM diagnostic criteria of PM were as follows: 1) clinical signs and symptoms (persistent or recurrent fever, bulging fontanelles, neck stiffness or painful neck, vomiting, headache, altered mental status, seizures, or focal neurologic signs) that comply with meningitis; 2) CSF examination revealing at least one of the following (turbid appearance; CSF leukocytosis (>100 cells/μL); leukocytosis (10–100 cells/μL AND either an elevated protein (>1.0g/L) or decreased glucose (<2.2 mmol/L). If CSF protein and glucose results are not available, diagnose using the first 2 conditions (turbid appearance or leukocytosis >100 cells/μL). 3) *S. pneumoniae* was identified by microbiological testing (positive blood or CSF culture, positive CSF antigen detection, positive CSF mNGS testing) or Gram staining and microscopy of the CSF.

#### Inclusion criteria

The enrolled patients met the following three criteria.

(1) The admission date was between January 1, 2019, and December 31, 2020;
(2) age<15 years of age;
(3) hospitalized children with PM diagnosis in selected hospitals

#### Exclusion criteria

Patients were excluded from the study if cranial computed tomography (CT) and magnetic resonance imaging (MRI) were not performed.

### Data collection

Based on the International Classification of Diseases Tenth Revision diagnostic codes and Systematized Nomenclature of Medicine codes, these cases were identified by retrieving the electronic medical records for the discharge diagnoses “acute purulent meningitis” or “acute bacterial meningitis” or “central nervous system infection” or “intracranial infection” or “meningitis” in each collaborating hospital from January 2019 to December 2020. Only the first medical record was included for analysis if the patient was hospitalized multiple times for the same diagnosis. Medical records, laboratory, and microbiological data of all enrolled cases were systematically assessed and recorded by locally trained pediatric researchers using a standard case report form and entered into the Good Clinical Data Management System.

We retrospectively collected data including baseline information on demographics, clinical characteristics, the time of admission and discharge, craniofacial and spinal anatomical abnormalities associated with central nervous system infection, causative microorganisms, laboratory findings, cranial imaging, treatment, antibiotic susceptibility test results, and outcomes.

### Laboratory methods analysis

All enrolled cases with PM diagnosis underwent LP after admission and cerebrospinal fluid (CSF) examinations were conducted, encompassing leukocyte and red blood cell count, differential leukocyte count, CSF biochemical, CSF culture, Gram staining and microscopy), as well as blood routine test, blood cultures, and cranial imaging. CSF and blood specimens were cultured, and *S. pneumoniae* was identified using standard methods as previously described. Microbiological specimens were cultured by BACT/ALERT 3D 240 automatic blood culture instrument (Mérieux, France), and *S. pneumoniae* were identified by the automatic bacterial identification system (VITEK Compact 2, France) at each surveillance center. Drug susceptibility testing of *S. pneumoniae* to penicillin was supplemented with the *E*-test method (Sangji et al., 2012). The culture and identification procedures are followed following the National Clinical Laboratory Procedures (Hong et al., 2015). Normal reference ranges of CSF parameters adhered to the guidelines outlined in the National Clinical Laboratory Procedures (Hong et al., 2015). In this study, antigen detection and molecular biological detection methods were selectively employed in only some of the PM cases that were in serious status requiring earlier identification of pathogens or patients with empirical antibiotics therapy failed or patients with negative initial CSF Gram stains whose CSF and(or) blood cultures at 72 h incubation were negative (including BinaxNOW for Streptococcus pneumoniae detection and mNGS).

The interpretation of positive CSF mNGS results followed the guidelines established by the Expert Consensus on the Use of Cerebrospinal Fluid Metagenomic next-generation sequencing for Infectious Diseases of the Central Nervous System (Infectious Diseases and Cerebrospinal Fluid Cytology Group of the Neurology Section of the Chinese Medical Association, 2021).

### Identification of *S. pneumoniae* and antibiotic susceptibility test

The identification of *S. pneumoniae* isolates and antibiotic susceptibility tests were conducted by an automatic bacterial identification system (VITEK Compact, France) or Optochin Discs (OXOID, UK) according to the National Guide to Clinical Laboratory Procedures. Antimicrobial susceptibility results of *S. pneumoniae* isolates were interpreted according to the standards of Clinical and Laboratory Standards Institute (Clinical and Laboratory Standards Institute, 2021).

### Clinical outcomes and relevant definitions

We defined the clinical situation on the discharge day as the clinical outcome. “Cured” was characterized by relief of clinical symptoms and signs, negative CSF detect results, and the absence of cranial imaging complications during hospitalization (Sun, 2002). “Disease improvement” was considered as relief of clinical symptoms and signs, negative CSF culture, normalization of blood inflammatory indicators, approximately normal CSF leukocyte count, CSF protein and (or) glucose level not returned to normal level, and no progress in cranial imaging complications during hospitalization (Sun, 2002). “Unhealed” was defined as no improvement in clinical symptoms, abnormal CSF test results, and the presence of neurological complications observed on cranial imaging. “Death” was defined as a fatality occurring during hospitalization or within 3 days of discharge from the hospital after the cessation of treatment. Relapse was defined as the recurrence of bacterial meningitis caused by the same pathogenic bacteria after the completion of antimicrobial therapy from the initial episode (Subspecialty Group of Neurology et al., 2019). Adverse outcomes included unhealed, relapse, death, being discharged automatically, abandoned treatment, or being transferred to other hospitals for further treatment.

Predisposing factors of PM are underlying diseases and conditions that predispose a person to bacterial meningitis, including cerebrospinal fluid nasal/otorrhea, head trauma, intracranial or ear malformation, previous history of bacterial meningitis, perforation of the tympanic membrane, sacrococcygeal furs and cochlear implants, organ transplantation, and tumor-related diseases, basic metabolic disorders, congenital organ malformations, hereditary diseases, chronic diseases (van de Beek et al., 2016). Extracranial infectious diseases included pneumonia, mastoiditis, sinusitis, and otitis media (Infection Group of the Chinese Society of Pediatrics et al., 2018).

### Statistical analysis

SPSS 19.0 software (IBM Corporation, Armonk, NY, USA) was used for statistical analysis. Categorical data were expressed in frequency and percentage, and the chi-square test and Fisher’s exact test were used to analyze the statistical difference. Continuous data were expressed as median (Q1, Q3) for non-normal distribution and Kruskal–Wallis H-test was used to analyze the statistical difference, with the *P* value set at less than 0.05 with statistical significance.

## Results

### Baseline clinical characteristics

**Figure 1:**
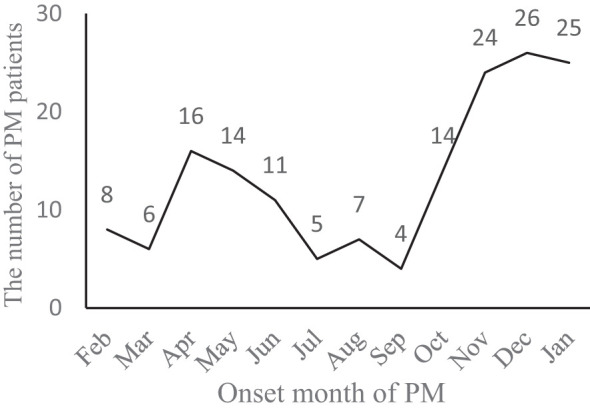
Number of PM patients in different months.

During Jan 1, 2019, and Dec 31, 2020, 167 children (<15 years old) with PM were enrolled for screening. 7 cases (4.2%) were excluded due to hospital-acquired, post-surgical, post-traumatic meningitis and two episodes for one person. A total of 160 PM patients met the inclusion criteria, including 105 (65.6%) in 2019 and 55 (34.4%) in 2020. There were 82 cases (51.3%) in East China, 30 cases (18.8%) in Southwest China, 15 cases (9.4%) in North China, 14 cases (8.8%) in Northwest China, 10 cases (6.3%) in Central China, 7 cases (4.4%) in Northeast China and 2 cases (1.3%) in South China. Among 160 PM children, 103 (64.4%) were males and 57 (35.6%) were females, with a male-to-female ratio of 1.8:1. The median age at presentation was 1 year and 3 months, with cases distributed as follows: 1 case (0.6%) under 28 days old, 5 cases (3.1%) between 28 days and 3 months old, 63 cases (39.4%) between 3 months and 1 year old, 46 cases (28.8%) aged 1 to under 3 years, 28 cases (17.5%) aged 3 to under 5 years, and 17 cases (10.6%) aged 5 to under 15 years. The median hospitalization time was 23 (17,30) days, and the hospitalization cost was 42 (28, 62) thousand yuan. In 2020, both the hospitalization time and cost were significantly lower than those in 2019 (Z=-3.113, -3.469; *P*=0.002, 0.001) (**Table 1**). The onset time of PM formed two peaks, with the highest incidence in November, December, and January of the subsequent year, followed by another peak in April, May, and June (**Figure 1**).

**Table 1:** Comparison of clinical data among PM children in different years [n (%)].

Characteristic	2019 (n=105)	2020 (n=55)	χ^2^/Z	*P* value
Demographics				
Median age (m)*	14 (7.3, 40.5)	16.3 (7.4, 45.0)	0.381	0.703
Male	70 (66.7)	33 (60.0)	0.699	0.403
Hospitalization time (d)^*^	24 (1, 100)	20 (0, 63)	-3.113	0.002
Hospitalization cost (×10^4^ yuan)	4.7 (0.9, 3.4)	3.3 (0.2, 14.2)	-3.469	0.001
Extracranial infectious diseases	75 (71.4)	38 (69.1)	0.095	0.758
Predisposing factors of PM	34 (32.4)	21 (38.2)	0.538	0.463
Symptoms and signs of presentation				
Fever	98 (93.3)	49 (89.1)	0.87	0.351
Vomit	42 (40.0)	19 (34.5)	0.455	0.5
Altered mental status	35 (33.3)	12 (21.8)	2.307	0.129
Neck stiffness	28 (26.7)	11 (20.0)	0.87	0.351
Convulsion	21 (20.0)	10 (18.2)	0.076	0.782
Bulging fontanelles	20 (19.0)	9 (16.4)	0.175	0.676
Headache	12 (11.4)	8 (14.5)	0.321	0.571
Critical presentation on admission				
Respiratory failure	20 (19.0)	3 (7.9)	5.418	0.020
Coma	17 (16.2)	3 (5.5)	3.804	0.048
Mechanical ventilation	11 (10.5)	2 (3.6)	1.439	0.230
Septic shock	6 (5.7)	1 (1.8)	0.544	0.461
*S. pneumoniae* detection results				
Positive CSF culture	62 (59.0)	33 (60.0)	0.014	0.907
Positive blood culture	41 (39.0)	16 (29.1)	1.56	0.212
Positive culture of CSF and blood	20 (19.0)	5 (9.1)	2.714	0.099
Positive CSF mNGS^※^	22 (37.9)	13 (44.8)	0.382	0.536
Positive CSF antigen detection^§^	14 (26.4)	7 (28.0)	0.022	0.883
Intracranial Imaging Complications^▲^				
Subdural effusion and (or) empyema	31 (29.5)	12 (21.8)	1.091	0.296
Hydrocephalus	18 (17.1)	6 (10.9)	1.1	0.294
Brain abscess	18 (17.1)	5 (9.1)	1.901	0.168
cerebral hemorrhage	7 (6.7)	1 (1.8)	0.911	0.340
Other cerebrovascular diseases^★^	10 (9.5)	3 (5.5)	0.348	0.555
Clinical outcome				
Cured	20 (19.0)	16 (29.1)	2.088	0.148
Disease improvement	73 (69.5)	33 (60.0)	1.464	0.226
Adverse outcomes^#^	12 (11.4)	6 (10.9)	0.01	0.921

PM, pneumococcal meningitis; CSF, cerebrospinal fluid; mNGS, metagenomic next-generation sequencing; m, month; d, day; *indicators were described as Median (Q1, Q3), and others were described as “number (%)”; ^※^87 PM children were tested for mNGS; ^§^78 PM children were tested for S. pneumoniae antigen; **^▲^** Intracranial Imaging Complications, subdural effusion and (or) pus, hydrocephalus, brain abscess, cerebral hemorrhage, and other cerebrovascular diseases on Magnetic Resonance imaging and (or) CT during hospitalization; ^★^Other cerebrovascular diseases included Encephalomalacia in 7 cases and Encephalatrophy and cerebral infarction in 3 cases each, and some children had more than one cerebrovascular disease at the same time; ^#^Adverse outcomes included 5 cases each of death and unhealed during hospitalization, 6 cases of automatic discharge and abandonment of treatment, and 2 cases of relapse.

### Predisposing factors and extracranial infectious diseases in PM cases

55 cases (34.4%) had one or more predisposing factors for bacterial meningitis, including 17 cases (10.6%) of CSF nasal/otorrhea, 13 cases (8.1%) of cranial trauma, 12 cases (7.5%) of intracranial or ear malformation, 9 cases (5.6%) of previous history of bacterial meningitis, 6 cases of leukemia. Of these, 1 case each (0.6%) was tympanic membrane perforation, sacrococcygeal fur sinus, cochlear implantation, hemophagocytic syndrome, and post-liver transplantation. Seven children had more than one predisposing factor for bacterial meningitis.

113 patients (70.6%) had one or more extracranial infections at the initial stage of PM, including 81 patients (50.6%) with pneumonia, 29 with mastoiditis (18.1%), 23 with sinusitis (14.4%) and 19 with otitis media (11.9%). There were no significant differences in the proportion of predisposing factors of bacterial meningitis and extracranial infectious diseases among different onset years (χ^2 ^= 0.397, 0.095; *P*=0.529, 0.758) (**Table 1**).

### Clinical presentation

The main clinical manifestations of PM children are presented in (**Table 1**). Fever (147 cases, 91.9%) was the most common symptom. 33 patients (20.6%) were hospitalized with a critical presentation on admission, including respiratory failure (23 cases, 14.4%), coma (20 cases, 12.5%), mechanical ventilation (13 cases, 8.1%), and septic shock (7 cases, 4.3%). PM critical presentations were predominant in the less than 1-year-old group (21 cases, 63.6%), including 12 cases of coma, 12 cases of mechanical ventilation, and 6 cases of septic shock. Three (1.9%) children were admitted with multiple organ failure, all of whom were less than 3 years of age. One child with PM developed a cerebral hernia and died on the day of admission. The proportion of critical presentations such as respiratory failure and coma in the 2020 group was significantly lower than that in the 2019 group (χ^2 ^= 5.418,3.804; *P*=0.020, 0.048) as shown in (**Table 1**).

### *S. pneumoniae* testing samples and results

*S. pneumoniae* detection samples and results are shown in (**Table 1**). Of the 35 children with positive CSF mNGS tests, the positive rates of cerebrospinal fluid cultures and blood cultures were 10.3% (87 PM cases performed mNGS test.) and 5.7%, respectively; the rate of both blood and CSF cultures was 1.1%, and the rate of positive results for CSF mNGS only was 25.3%. Among the 21 children with positive *S. pneumoniae* antigen test in CSF, the positive rate of CSF culture and blood culture was each 10.3% (78 PM cases performed *S. pneumoniae* antigen test.), the positive rate of both blood and CSF culture was 3.8%, and the positive rate of CSF *S. pneumoniae* antigen test result was only 6.4%.

### Intracranial imaging complications in PM children

Intracranial complications were identified through cranial magnetic resonance imaging and (or) cranial CT scans during hospitalization in 69 (43.1%) children, of which 17 (25%) had more than 1 intracranial imaging complication. The common intracranial complications were subdural effusion and (or) empyema in 43 cases (26.9%), hydrocephalus in 24 cases (15.0%), brain abscess in 23 cases (14.4%), cerebral hemorrhage in 8 cases (5.0%), and other cerebral vascular disorders (including encephalomalacia, cerebral infarction, and encephalatrophy) in 13 cases (8.1%). Subdural effusion and (or) empyema and hydrocephalus manifested predominantly in children under the age of one in 90.7% and 83.3%, respectively, which was shown in (**Table 1**).

### Adverse outcome

The rates of cured and disease improvement in PM cases were 22.5% and 66.3%, respectively. 18 (11.3%) children had adverse outcomes, including 5 (3.1%) children who died during hospitalization (two patients were <1 year old, one patient 2 years old, and two patients >5 years old) and 4 early deaths (within 1 week of hospitalization); two children had relapses (one case within 3 weeks and 1 case after 3 weeks, both in 2019); five cases not cured, and six cases of automatic discharge and (or) abandonment of treatment.

### Laboratory parameters

The initial CSF leukocyte count was documented in 147 (91.9%), the initial glucose concentration of CSF in 156 (97.5%), and the initial protein concentration of CSF in 152 (95.0%) of the 160 children diagnosed with PM.

The initial leukocyte counts of CSF were normal in two children, both of whom were younger than 1 year of age (10 months of age and 5 months and 19 d, respectively). CSF cultures of one child were positive for *S. pneumoniae* and of one child were positive for both blood and cerebrospinal fluid *S. pneumoniae* cultures. Two cases with normal initial leukocyte counts in CSF had a decrease in the initial glucose concentration of CSF (1.1 mmol/L, 1.0 mmol/L, respectively), and an increase in the initial protein concentration of CSF (3.0 and1.6 g/L, respectively).

Among the 20 children with an initial CSF glucose concentration of ≥2.8 mmol/L, 12 (60.0%) exhibited positive CSF cultures, while three (15.0%) had positive results in both blood and CSF cultures, as well as in CSF mNGS. Additionally, both CSF culture and CSF mNGS were positive in 1 case, and only CSF mNGS was positive in 1 case (5.0%). The initial CSF leukocyte count was elevated in 20 cases, of which 10 cases were >1000×106/L, 2 cases were >500∼1000×106/L, 5 cases were 100∼500×106/L, and 3 cases were <100×106/L; the initial CSF protein concentration was >1000 mg/L in 10 cases, (450∼1000) mg/L in 4 cases, and normal in 6 cases. The initial CSF parameters of the children and pre-discharge from the hospital are shown in (**Table 2**).

**Table 2:** Initial and pre-discharge CSF parameters in PM cases [n (%)].

parameters	Initial	Pre-discharge
Leukocyte count of CSF (×10^6^/L)	147(91.9)^★^	142(88.8)^★^
>1000	63(39.4)	5(3.5)
Normal∼1000	82(51.3)	111(78.1)
Normal**^a^**	2(1.3)	26(18.3)
glucose concentration of CSF (mmol/L)	156(97.5)^★^	149(93.1)^★^
<1.1	56(35.0)	0
≥1.1 ~ < 2.2	59(36.9)	18(11.3)
≥ 2.2 ~ < 2.8	21(13.1)	64(40.0)
≥ 2.8	20(12.5)	67(41.9)
Protein concentration of CSF (g/L)	152(95.0)^★^	136(85.0)^★^
>1.5	87(54.4)	20(12.5)
1∼1.5	29(18.1)	14(8.8)
<1.0	36(22.5)	102(63.8)

*Injury of lumbar puncture leads to the inability to extract or contaminate CSF samples. ^a^ non- neonate: <5×10^6^/L; neonate: <20×10^6^/L.

PM, pneumococcal meningitis.

### Antimicrobial susceptibility analysis of *S. pneumoniae*

Among 157 *S. pneumoniae* strains isolated, only 91, *S. pneumoniae* antimicrobial susceptibility data were registered (84 from CSF, 7 from blood) (**Table 3**). Seven blood-derived *S. pneumoniae* strains were determined for antimicrobial susceptibility according to the breakpoint of parenteral administration of meningitis strain. We found that 68 *S. pneumoniae* isolates underwent penicillin susceptibility testing: 11 (16.2%) with susceptibility and 57 (83.8%) with resistance to penicillin. The susceptibility rates of *S. pneumoniae* to levofloxacin, moxifloxacin, rifampicin, and chloramphenicol were 81.5%, 82.4%, 96.2%, and 91.3%. The susceptibility rates of *S. pneumoniae* to cefotaxime, meropenem, and ceftriaxone were 56.1%, 51.1%, and 63.5, respectively. No *S. pneumoniae* isolates were resistant to vancomycin, linezolid, or ertapenem. *S. pneumoniae* were completely resistant to erythromycin.

**Table 3:** The antimicrobial susceptibility test of *S. pneumoniae* isolated from PM patients. [strain (%)].

Antimicrobial drugs	Number of *S. pneumoniae*	Susceptible	not- susceptible		
			Intermediate	Resistant	total
Penicillin	68	11 (16.2)	NA	57 (83.8)	57 (83.8)
Ceftriaxone	52	33 (63.5)	15 (28.8)	4 (7.7)	19 (36.5)
Cefotaxime	41	23 (56.1)	10 (24.4)	8 (19.5)	18 (43.9)
Meropenem	45	23 (51.1)	14 (31.1)	8 (17.8)	22 (48.9)
Cotrimoxazole	49	21 (42.9)	9 (18.4)	19 (38.7)	25 (57.1)
Levofloxacin	27	22 (81.5)	2 (7.4)	3 (11.1)	5 (18.5)
Chloramphenicol	23	21 (91.3)	NA	2 (8.7)	2 (8.7)
Clindamycin	17	1 (1/17)	0(0)	16 (16/17)	16 (16/17)
Tetracycline	21	1 (4.8)	2 (9.5)	18 (85.7)	20 (95.2)
Erythromycin	31	0 (0)	0(0)	31 (100.0)	31 (100.0)
Vancomycin	75	75 (100.0)	0(0)	0(0)	0(0)
Linezolid	56	56 (100.0)	0(0)	0(0)	0(0)
Ofloxacin	31	31 (100.0)	0(0)	0(0)	0(0)
Ertapenem	6	6 (6/6)	0(0)	0(0)	0(0)
Moxifloxacin	17	14 (14/17)	0(0)	3 (3/17)	3 (3/17)
Rifampicin	26	25 (96.2)	0(0)	1 (3.8)	1 (3.8)

PM, pneumococcal meningitis; NA, not applicable

## Discussion

PM is associated with a high mortality and morbidity rate, along with significant and serious long-term effects on survivors (Collaborators. GCoD., 2018; Chen et al., 2011; Mook-Kanamori et al., 2011). Global Burden of Disease Study in 2017 shows PM accounts for 40.1% of Bacterial Meningitis in Infants and Children aged 1-59 months (Wright et al., 2021). World Health Organization reports that the global mortality rate of PM is 12.2% (Nakamura et al., 2021). In Europe, the mortality rate of PM is 5.5% to 16% (Polkowska et al., 2021; Buchholz et al., 2016). The mortality rate of PM in China is 23.5%-46.2% (Xu et al., 2021). The incidence of neurologic long-term sequelae of PM is 25% to 50% (O'Brien et al., 2009; GBD 2019 Meningitis Antimicrobial Resistance Collaborators, 2023). Analyzing the clinical epidemiological characteristics of PM cases can identify and diagnose PM at an early stage and provide a theoretical basis for the rational use of antimicrobials in the clinic.

Two multicenter studies on bacterial meningitis in children conducted in China during 2014-2016 and 2019-2020 (Guo et al., 2016; Wang et al., 2022) indicated that *S. pneumoniae* was the primary causative agent in children older than 3 months, constituting 46.9% and 46.7% of cases, respectively. This study revealed that the peak age of onset of PM in children occurs between 3 months of age and less than 3 years of age, which was in agreement with the 2012 World Health Organization report (Publication., 2012). In this study, the incidence of PM formed 2 peaks, and the incidence season was most frequent in November, December, and January of the following year, followed by April, May, and June, which was associated with the high incidence of respiratory infectious diseases in winter and spring, which was consistent with other reports (Fricke et al., 2021; Fang et al., 2018).

PM typically results from *S. pneumoniae* and is commonly associated with bloodstream infections such as bacteremia and sepsis, or directly involves the meninges through adjacent tissue infections (Mook-Kanamori et al., 2011; Gil et al., 2022). Respiratory viral infections such as influenza virus, respiratory syncytial virus, and adenovirus are high-risk factors for *S. pneumoniae* causing bloodstream infections (Ampofo et al., 2008; Principi et al., 2023). Since the outbreak of novel coronavirus pneumonia in December 2019, non-pharmacological interventions such as stay-at-home orders, maintaining social distancing, online courses for students, mask-wearing, and hand hygiene have been implemented to mitigate the outbreak of novel coronavirus pneumonia, which has been effective in preventing the novel coronavirus pneumonia while reducing the chances of other respiratory micro-organisms infections in children (Losier et al., 2023; Chiu et al., 2020),. Additionally, they have objectively restricted the transfer of complicated and critically ill cases to higher-level hospitals (Fricke et al., 2021). The study indicates that PM hospitalizations and hospitalization rates decreased after the novel coronavirus pneumonia epidemic compared with the pre-epidemic period. Additionally, the proportion of children admitted with PM presenting critical symptoms such as respiratory failure and coma, hospitalization stay, and hospitalization cost were significantly lower than those observed in 2019.

With widespread vaccination, effective anti-infective measures, and comprehensive treatment programs, the mortality rates associated with bacterial meningitis have significantly declined, showing a 21.0% reduction from 1990 to 2016 (Collaborators. GCoD., 2018). The most notable aspect of this study is the lower case fatality rate of PM, which was only 3.1%, significantly lower than the results of a national multicenter clinical study of PM conducted from 2013-2017, in which 16.8% of children died (Wang et al., 2019). The incidence of intracranial complications in this study was 43.1%, which was lower than the results of the 2013 2017 PM multicenter study (49.6%). The same trend was observed for systemic complications. Therefore, the reduction in both intracranial complications, and potential systemic complications, coupled with the implementation of preventive and control measures taken to reduce the transmission of novel coronaviruses may be associated with lower PM morbidity and mortality in this study.

Common predisposing factors for PM include CSF nasal/otorrhea leakage, cranial trauma, intracranial or ear malformation, previous history of bacterial meningitis, tympanic membrane perforation, sacrococcygeal pilonidal sinus and cochlear implantation, post-transplantation, and tumor-related diseases (Infection Group of the Chinese Society of Pediatrics et al., 2018). The proportion of predisposing factors for PM in this study was 34.3%, which is in agreement with the results of previous studies generally (21.3% to 34.0%) (Hénaff et al., 2017). In this study, there were two relapses, one within 3 weeks of discharge and one 3 weeks after discharge; one case of CSF nasal leakage, and one case of critical condition after relapse without identification of the underlying etiology. According to the recommendations for the management of bacterial meningitis, after confirming the diagnosis of meningitis, it is necessary to verify the presence of predisposing factors and extracranial infectious diseases in the child (Subspecialty Group of Neurology et al., 2019).

CSF routine examination and culture are essential in the diagnostic workup of all children with suspected bacterial meningitis (Brouwer et al., 2012). Currently, the rate of positive CSF and(or) sterile body fluid cultures for *S. pneumoniae* is relatively low (Prasad and Sahu, 2011). Molecular techniques (antigen detection, nucleic acid detection, and mNGS) are effective in improving the detection of pathogens in bacterial meningitis (Wilson et al., 2019; Miller et al., 2019). In this study, the number of *S. pneumoniae* detected by mNGS and antigen detection methods was 22 (13.8%) and 8 (5.0%), respectively. Molecular techniques such as mNGS and antigen detection can effectively improve the detection rate of pathogens in PM, especially when bacterial meningitis is highly suspected in clinic but pathogens are unclear. Pathogenetic diagnosis has always been the most important in the diagnosis of clinical infectious diseases, and the shift from traditional culture to molecular biology pathogen diagnosis requires a shift in thinking on the part of clinicians and clinical biologists. Therefore, clinicians need to be familiar with new advances in this field in order to better apply microbiological tests so that children can receive a definitive pathogenetic diagnosis earlier, be treated accurately, and have an improved disease prognosis.

Drug resistance of *S. pneumoniae* is a global public health problem and a serious threat to the health of children (Kim et al., 2012). *S. pneumoniae* strains in children with PM in China are extremely insensitive to penicillin, and the penicillin resistance rate of *S. pneumoniae* to penicillin in this group of cases was 83.8%, which is similar to the penicillin resistance rate of meningitis strains in a multicenter meningitis study from 2013 to 2017 (80.4%,111/138 strains) (Wang et al., 2019), suggesting that penicillin can’t be the first-line medication in the treatment of PM. Meanwhile, the insensitivity rates of *S. pneumoniae* isolates to ceftriaxone and cefotaxime were 36.5% and 43.9%, respectively, which suggested that *S. pneumoniae* strains are highly resistant to β-lactam antimicrobial drugs, which may be related to the wide application of β-lactam antimicrobial drugs in clinical practice.

This study was a retrospective analysis. According to the established PM diagnostic criteria, the local data diagnosed as bacterial meningitis were re-evaluated and summarized to increase the reliability of the study results. This study also has some limitations: 1) All cases were from tertiary hospitals, and some individual critically ill children failed to undergo lumbar puncture and imaging in each of the collaborating hospitals and could not be included in the study, which affected the assessment of the incidence of PM, morbidity and mortality, and imaging complications. 2) The annual number of cases in each of the collaborating hospitals is low, especially in 2020 prevention and control measures taken to mitigate the spread of novel coronaviruses in 2020, which affected the transfer of complex cases to higher-level hospitals, and there may have been case selection bias; 3) Data such as the onset of PM, disease progression, antibiotic medication, *S. pneumoniae* serotypes, and vaccination were not collected and analyzed; 4)PM cases with complications were not followed up further for long-term outcomes. These limitations need to be improved in the course of subsequent continuous monitoring of PM in order to obtain more complete and accurate monitoring results.

## Data availability statement

The raw data supporting the conclusions of this article will be made available by the authors, without undue reservation.

## Ethics statement

The studies involving humans were approved by the Committee of Children’s Hospital of Zhejiang University School of Medicine (2019-IRB-094). The studies were conducted in accordance with the local legislation and institutional requirements. The human samples used in this study were acquired from primarily isolated as part of your previous study for which ethical approval was obtained. Written informed consent for participation was not required from the participants or the participants’ legal guardians/next of kin in accordance with the national legislation and institutional requirements.

## Author contributions

CW: Project administration, Data curation, Investigation, Writing – original draft, Writing – review & editing. HX: Data curation, Formal analysis, Writing – review & editing. GL: Data curation, Formal analysis, Writing – review & editing. JL: Data curation, Formal analysis, Writing – review & editing. HY: Data curation, Formal analysis, Writing – review & editing. BC: Data curation, Formal analysis, Writing – review & editing. GZ: Data curation, Formal analysis, Writing – review & editing. MS: Data curation, Formal analysis, Writing – review & editing. LD: Data curation, Formal analysis, Writing – review & editing. ZX: Data curation, Formal analysis, Writing – review & editing. LH: Data curation, Formal analysis, Writing – review & editing. HL: Data curation, Formal analysis, Writing – review & editing. SS: Data curation, Formal analysis, Writing – review & editing. YC: Project administration, Supervision, Writing – review & editing.

## Collaborators

Corporate Authors of Chinese Pediatric Bacterial Meningitis Surveillance (CPBMS) Study Group: Dong Wang, Huiling Deng, Songting Bai, Qingwen Shan, Chunhui Zhu, Jianmei Tian, Jianhua Hao, Aiwei Lin, Daojiong Lin, Jinzhun Wu, Xinhua Zhang, Qing Cao, Zhongbin Tao, Yuan Chen, Guolong Zhu, Ping Xue, Zhengzhen Tang, Xuewen Su, Zhenghai Qu, Shiyong Zhao, Lin Pang. The CPBMS team: the Children’s Hospital of Zhejiang University School of Medicine, National Clinical Research Center for Child Health, National Children’s Regional Medical Center, Children’s Hospital of Chongqing Medical University, Beijing Children’s Hospital, Capital Medical University, National Center for Children’s Health, Research Unit of Critical Infection in Children, Chinese Academy of Medical Sciences, 2019RU016, Hunan Children’s Hospital, the Children’s Hospital of Fudan University, Anhui Province Children’s Hospital, Children’s Hospital of Nanjing Medical University, West China Second University Hospital, Sichuan University/West China Women’s and Children’s Hospital, Shanxi Children’s Hospital, the 2nd Affiliated Hospital and Yuying Children’s Hospital of Wenzhou Medical University, Xinhua Hospital Affiliated to Shanghai Jiao Tong University School of Medicine, the First Hospital of Jilin University, the Affiliated Children’s Hospital of Xi’an Jiaotong University, Xi’an Central Hospital, the First Affiliated Hospital of Zhengzhou University, the First Affiliated Hospital of Guangxi Medical University, Jiangxi Provincial Children’s Hospital, Children’s Hospital of Soochow University, Kaifeng Children’s Hospital, Children’s Hospital Affiliated to Shandong University, Hainan Women and Children’s medical center, Women and Children’s Hospital, School of Medicine, Xiamen University, Shanghai Children’s Medical Center, National Children’s Medical Center, Shanghai Jiaotong University School of Medicine, the First Hospital of Lanzhou University, the Second Hospital of Hebei Medical University, the Women’s and Children’s Hospital of Qinghai Province, Taiyuan Maternal and Child Health Care Hospital, the First People’s Hospital of Zunyi, Inner Mongolia People’s Hospital, the Affiliated Hospital of Qingdao University, Hangzhou Children’s Hospital, Beijing Ditan Hospital, Capital Medical University, Tongji Hospital, Tongji Medical College of Huazhong University of Science and Technology.

## References

[B1] Ampofo K. Bender J. Sheng X. Korgenski K. Daly J. Pavia A. T.. (2008). Seasonal invasive pneumococcal disease in children: role of preceding respiratory viral infection. Pediatrics. 122, 229–237. doi: 10.1542/peds.2007-3192 18676537

[B2] Brouwer M. C. Thwaites G. E. Tunkel A. R. van de Beek D. (2012). Dilemmas in the diagnosis of acute community-acquired bacterial meningitis. Lancet. 380, 1684–1692. doi: 10.1016/S0140-6736(12)61185-4 23141617

[B3] Buchholz G. Koedel U. Pfister H. W. Kastenbauer S. Klein M. (2016). Dramatic reduction of mortality in pneumococcal meningitis. Crit. Care 20, 312. doi: 10.1186/s13054-016-1498-8 27716447 PMC5045860

[B4] Chen Y. Deng W. Wang S. M. Mo Q. M. Jia H. Wang Q.. (2011). Burden of pneumonia and meningitis caused by Streptococcus pneumoniae in China among children under 5 years of age: a systematic literature review. PloS One 6 (11), e27333. doi: 10.1371/journal.pone.0027333 22110628 PMC3217934

[B5] Chiu N. C. Chi H. Tai Y. L. Peng C. C. Tseng C. Y. Chen C. C.. (2020). Impact of wearing masks, hand hygiene, and social distancing on influenza, enterovirus, and all-cause pneumonia during the coronavirus pandemic: retrospective national epidemiological surveillance study. J. Med. Internet Res. 22 (8), e21257. doi: 10.2196/21257 32750008 PMC7471891

[B6] Clinical and Laboratory Standards Institute. (2021). Performance standards for antimicrobial susceptibility testing, M100-S31. (Wayne, PA: CLSI). 10.1128/JCM.00213-21 PMC8601225 34550809

[B7] Collaborators GCoD (2017). Global, regional, and national age-sex specific mortality for 264 causes of death, 1980-2016: a systematic analysis for the Global Burden of Disease Study 2016. Lancet. 390, 1151–1210. 28919116 10.1016/S0140-6736(17)32152-9 PMC5605883

[B8] Collaborators. GCoD. (2018). Global, regional, and national burden of meningitis, 1990-2016: a systematic analysis for the Global Burden of Disease Study 2016. Lancet Neurol. 17, 1061–1082. doi: 10.1016/S1474-4422(18)30387-9 30507391 PMC6234314

[B9] Fang C. Chen X. J. Zhou M. M. Chen Y. H. Zhao R. Z. Deng J. K.. (2018). [Clinical characteristics and antimicrobial resistance of pneumococcal infections from 9 children's hospitals in 2016]. Zhonghua Er Ke Za Zhi. 56 (8), 582–586. doi: 10.3760/cma.j.issn.0578-1310.2018.08.005 30078238

[B10] Fricke L. M. Glöckner S. Dreier M. Lange B. (2021). Impact of non-pharmaceutical interventions targeted at COVID-19 pandemic on influenza burden - a systematic review. J. Infect. 82, 1–35. doi: 10.1016/j.jinf.2020.11.039 PMC9183207 33278399

[B11] GBD 2016 Brain and Other CNS Cancer Collaborators. (2019). Global, regional, and national burden of brain and other CNS cancer, 1990-2016: a systematic analysis for the Global Burden of Disease Study 2016. Lancet Neurol. 18 (4), 376–393. doi: 10.1016/S1474-4422(18)30468-X 30797715 PMC6416167

[B12] GBD 2016 Neurology Collaborators. (2019). Global, regional, and national burden of neurological disorders, 1990-2016: a systematic analysis for the Global Burden of Disease Study 2016. Lancet Neurol. 18, 459–480. doi: 10.1016/S1474-4422(18)30499-X 30879893 PMC6459001

[B13] GBD 2019 Meningitis Antimicrobial Resistance Collaborators. (2023). Global, regional, and national burden of meningitis and its aetiologies, 1990-2019: a systematic analysis for the Global Burden of Disease Study 2019. Lancet Neurol. 22, 685–711. doi: 10.1016/S1474-4422(23)00195-3 37479374 PMC10356620

[B14] Gil E. Wall E. Noursadeghi M. Brown J. S. (2022). Streptococcus pneumoniae meningitis and the CNS barriers. Front. Cell Infect. Microbiol. 12, 1106596. doi: 10.3389/fcimb.2022.1106596 36683708 PMC9845635

[B15] Guo L. Y. Zhang Z. X. Wang X. Zhang P. P. Shi W. Yao K. H.. (2016). Clinical and pathogenic analysis of 507 children with bacterial meningitis in Beijing, 2010-2014. Int. J. Infect. Dis. 50, 38–43. doi: 10.1016/j.ijid.2016.07.010 27452172

[B16] Hénaff F. Levy C. Cohen R. Picard C. Varon E. Gras Le Guen C.. (2017). Risk factors in children older than 5 years with pneumococcal meningitis: data from a national network. Pediatr. Infect. Dis. J. 36, 457–461. doi: 10.1097/INF.0000000000001470 28403047

[B17] Hong S. Yusan W. Ziyu S. (2015). National guide to Clinical laboratory procedures. 5 (Beijing: People's Medical Publishing House).

[B18] Infection Group of the Chinese Society of Pediatrics Chinese Medical Association Editorial Board of the Chinese Journal of Pediatrics. (2018). [Diagnosis, treatment, prevention and control of Streptococcus pneumoniae diseases in children]. Zhonghua Er Ke Za Zhi. 56, 564–570. doi: 10.3760/cma.j.issn.0578-1310.2018.08.002 30078235

[B19] Infectious Diseases and Cerebrospinal Fluid Cytology Group of the Neurology Section of the Chinese Medical Association. (2021). Expert consensus on clinical application of metagenomic next−generation sequencing of cerebrospinal fluid in the diagnosis of infectious diseases of the central nervous system. Chin. J. Neurol. 54, 1234–1240. doi: 10.3760/cma.j.cn113694-20210730-00532

[B20] Kim S. H. Song J. H. Chung D. R. Thamlikitkul V. Yang Y. Wang H.. (2012). Changing trends in antimicrobial resistance and serotypes of Streptococcus pneumoniae isolates in Asian countries: an Asian Network for Surveillance of Resistant Pathogens (ANSORP) study. Antimicrob. Agents Chemother. 56 (3), 1418–1426. doi: 10.1128/AAC.05658-11 22232285 PMC3294909

[B21] Li C. Feng W. Y. Lin A. W. Zheng G. Wang Y. C. Han Y. J.. (2018). Clinical characteristics and etiology of bacterial meningitis in Chinese children >28 days of age, January 2014-December 2016: A multicenter retrospective study. Int. J. Infect. Dis. 74, 47–53. doi: 10.1016/j.ijid.2018.06.023 30100536

[B22] Losier A. Gupta G. Caldararo M. Dela Cruz C. S. (2023). The impact of coronavirus disease 2019 on viral, bacterial, and fungal respiratory infections. Clin. Chest Med. 44, 407–423. doi: 10.1016/j.ccm.2022.11.018 37085229 PMC9968485

[B23] Miller S. Naccache S. N. Samayoa E. Messacar K. Arevalo S. Federman S.. (2019). Laboratory validation of a clinical metagenomic sequencing assay for pathogen detection in cerebrospinal fluid. Genome Res. 29 (3), 831–842. doi: 10.1101/gr.238170.118 30992304 PMC6499319

[B24] Mook-Kanamori B. B. Geldhoff M. van der Poll T. van de Beek D. (2011). Pathogenesis and pathophysiology of pneumococcal meningitis. Clin. Microbiol. Rev. 24, 557–591. doi: 10.1128/CMR.00008-11 21734248 PMC3131058

[B25] Nakamura T. Cohen A. L. Schwartz S. Mwenda J. M. Weldegebriel G. Biey J. N. M.. (2021). The global landscape of pediatric bacterial meningitis data reported to the world health organization-coordinated invasive bacterial vaccine-preventable disease surveillance network, 2014-2019. J. Infect. Dis. 224 (12 Suppl 2), S161–s173. doi: 10.1093/infdis/jiab217 34469555 PMC8409679

[B26] O'Brien K. L. Wolfson L. J. Watt J. P. Henkle E. Deloria-Knoll M. McCall N.. (2009). Burden of disease caused by Streptococcus pneumoniae in children younger than 5 years: global estimates. Lancet. 374, 893–902. doi: 10.1016/S0140-6736(09)61204-6 19748398

[B27] Organization W. H. (2019). Pneumococcal conjugate vaccines in infants and children under 5 years of age: WHO position paper-February 2019. Weekly epidemiological Rec. 94, 85–104.

[B28] Organization WH. (2003) WHO-recommended standards for surveillance of selected vaccine preventable diseases. Available online at: http://www.measlesrubellainitiative.org/wp-content/uploads/2013/06/WHO-surveillance-standard.pdf (Accessed November 5, 2013).

[B29] Organization WH. (2021) Immunization coverage [EB/OL]. Available online at: https://www.who.int/news-room/fact-sheets/detail/immunization-coverage (Accessed 12-10, 2022).

[B30] Polkowska A. Rinta-Kokko H. Toropainen M. Palmu A. A. Nuorti J. P. (2021). Long-term population effects of infant 10-valent pneumococcal conjugate vaccination on pneumococcal meningitis in Finland. Vaccine. 39, 3216–3224. doi: 10.1016/j.vaccine.2021.02.030 33934915

[B31] Prasad K. Sahu J. K. (2011). Cerebrospinal fluid lactate: is it a reliable and valid marker to distinguish between acute bacterial meningitis and aseptic meningitis? Crit. Care 15, 104. doi: 10.1186/cc9396 21349143 PMC3222026

[B32] Principi N. Autore G. Ramundo G. Esposito S. (2023). Epidemiology of respiratory infections during the COVID-19 pandemic. Viruses. 15 (5), 1160. doi: 10.3390/v15051160 37243246 PMC10224029

[B33] Publication. W. (2012). Pneumococcal vaccines WHO position paper - 2012 - recommendations. Vaccine. 30, 4717–4718. doi: 10.1016/j.vaccine.2012.04.093 22621828

[B34] Sangji Y. Hui W. Xuzhuang S.. (2012). Consensus on clinical testing protocols for Streptococcus pneumoniae. Chin. J. Lab. Med. 35, 1066–1072.

[B35] Subspecialty Group of Neurology The Society of Pediatrics Chinese Medical Association. (2019). Expert consensus on diagnosis and treatment of community acquired bacterial meningitis in children. Zhonghua Er Ke Za Zhi. 57, 584–591. doi: 10.3760/cma.j.issn.0578-1310.2019.08.003 31352742

[B36] Sun C. (2002). Clinical disease diagnosis baiss criteria for cure and improvement. 2 (Beijing: People's Military Medical Press).

[B37] van de Beek D. Cabellos C. Dzupova O. Esposito S. Klein M. Kloek A. T.. (2016). ESCMID guideline: diagnosis and treatment of acute bacterial meningitis. Clin. Microbiol. Infect. 22 Suppl 3, S37–S62. doi: 10.1016/j.cmi.2016.01.007 27062097

[B38] Wang C. Y. Xu H. M. Deng J. K. Yu H. Chen Y. P. Lin A. W.. (2019). [A multicentric clinical study on clinical characteristics and drug sensitivity of children with pneumococcal meningitis in China]. Zhonghua Er Ke Za Zhi. 57 (5), 355–362. doi: 10.3760/cma.j.issn.0578-1310.2019.05.008 31060128

[B39] Wang C. Y. Xu H. M. Tian J. Hong S. Q. Liu G. Wang S. X.. (2022). [A multicenter epidemiological study of acute bacterial meningitis in children]. Zhonghua Er Ke Za Zhi. 60 (10), 1045–1053. doi: 10.3760/cma.j.cn112140-20220608-00522 36207852

[B40] Wilson M. R. Sample H. A. Zorn K. C. Arevalo S. Yu G. Neuhaus J.. (2019). Clinical metagenomic sequencing for diagnosis of meningitis and encephalitis. N Engl. J. Med. 380 (24), 2327–2340. doi: 10.1056/NEJMoa1803396 31189036 PMC6764751

[B41] Wright C. Blake N. Glennie L. Smith V. Bender R. Kyu H.. (2021). The global burden of meningitis in children: challenges with interpreting global health estimates. Microorganisms. 9 (2), 377. doi: 10.3390/microorganisms9020377 33668442 PMC7917636

[B42] Xu Y. Wang Q. Yao K. Dong F. Song W. Liu G.. (2021). Clinical characteristics and serotype distribution of invasive pneumococcal disease in pediatric patients from Beijing, China. Eur. J. Clin. Microbiol. Infect. Dis. 40 (9), 1833–1842. doi: 10.1007/s10096-021-04238-x 33786728

